# Physiological Consequences of Targeting 14-3-3 and Its Interacting Partners in Neurodegenerative Diseases

**DOI:** 10.3390/ijms232415457

**Published:** 2022-12-07

**Authors:** Akshatha Ganne, Meenakshisundaram Balasubramaniam, Nirjal Mainali, Paavan Atluri, Robert J. Shmookler Reis, Srinivas Ayyadevara

**Affiliations:** 1 Reynolds Institute on Aging, Department of Geriatrics, University of Arkansas for Medical Sciences, Little Rock, AR 72205, USA; 2 BioInformatics Program, University of Arkansas at Little Rock and University of Arkansas for Medical Sciences, Little Rock, AR 72205, USA; 3 Department of Biology, University of Arkansas, Fayetteville, AR 72701, USA; 4 Central Arkansas Veterans Healthcare Service, Little Rock, AR 72205, USA

**Keywords:** protein aggregation, Alzheimer’s disease, neurodegeneration, PPII drugs, 14-3-3, hexokinase

## Abstract

The mammalian 14-3-3 family comprises seven intrinsically unstructured, evolutionarily conserved proteins that bind >200 protein targets, thereby modulating cell-signaling pathways. The presence of 14-3-3 proteins in cerebrospinal fluid provides a sensitive and specific biomarker of neuronal damage associated with Alzheimer’s disease (AD), Creutzfeldt–Jakob disease (CJD), spongiform encephalitis, brain cancers, and stroke. We observed significant enrichment of 14-3-3 paralogs G, S, and Z in human brain aggregates diagnostic of AD. We used intra-aggregate crosslinking to identify 14-3-3 interaction partners, all of which were significantly enriched in AD brain aggregates relative to controls. We screened FDA-approved drugs in silico for structures that could target the 14-3-3G/hexokinase interface, an interaction specific to aggregates and AD. *C. elegans* possesses only two 14-3-3 orthologs, which bind diverse proteins including DAF-16 (a FOXO transcription factor) and SIR-2.1 (a sensor of nutrients and stress), influencing lifespan. Top drug candidates were tested in *C. elegans* models of neurodegeneration-associated aggregation and in a human neuroblastoma cell-culture model of AD-like amyloidosis. Several drugs opposed aggregation in all models assessed and rescued behavioral deficits in *C. elegans* AD-like neuropathy models, suggesting that 14-3-3 proteins are instrumental in aggregate accrual and supporting the advancement of drugs targeting 14-3-3 protein complexes with their partners.

## 1. Introduction

14-3-3 proteins comprise small families of paralogous proteins (seven in mammals, two in nematodes) that serve as modulators of critical signaling and regulatory pathways; e.g., via their interactions with transcription factors, including FOXO and TFEB [1]. The 14-3-3 protein family in mammals comprises seven 30-kDa proteins designated as β, γ, ε, τ, η, σ, and ζ paralogs (beta, gamma, epsilon, tau, eta, sigma, and zeta). These mammalian 14-3-3 genes, situated at seven distinct chromosomal loci, are expressed ubiquitously but are especially abundant in cerebral neurons [2]. They have especially high binding affinities for proteins containing phospho-serine or phospho-threonine and serve as both molecular chaperones and regulatory modulators of their numerous protein ligands [3]. They occur predominantly as homo- and heterodimers, forming complexes with over 200 other ligand proteins. Their highly disordered C-terminal and N-terminal domains [4] are generally stabilized by protein–protein interactions (PPIs) with their binding partners, through which they modulate signal transduction, apoptosis, cell-cycle progression, RNA transcription, and DNA replication [3,5]. 

Each 14-3-3 monomer contains a highly conserved and well-structured core region comprising nine alpha helices. Of these, helices 3, 5, 7, and 9 form a W- or L-shaped cluster with a concave amphipathic groove (combining hydrophilic and hydrophobic moieties) that interacts with ligand peptides, which are often (but not necessarily) phosphopeptides. Dimerization of 14-3-3 proteins entails interaction between helix H1 of one monomer and H3 and H4 of the other and may drive dimerization of their binding partners [6]. The final structural conformation of 14-3-3 paralogs is influenced by their interacting partners, believed to confer stable structures to the otherwise unstructured N- and C-terminal tails. Functions of 14-3-3 paralogs, in conjunction with their ligand proteins, span a wide range of enzymatic activities, subcellular localizations, and pathways [7]. Many binding partners of 14-3-3 contain RSX(pS/T)XP and/or RXXX(pS/T)XP consensus sequences, where X is any amino acid and (pS/T) indicates phosphoserine or phosphothreonine [8]. 

Expression patterns vary among the 14-3-3 paralogs. In mammals, 14-3-3E (ε, epsilon) protein is highly abundant in the brain, lymphoblasts, and adipocytes, while 14-3-3G (γ, gamma) is most highly expressed in the brain, skeletal muscle, heart, and embryonic stem cells. Both of these paralogs have been shown to oppose androgen biosynthesis [8]. The 14-3-3G (γ) paralog has been implicated in neurodegenerative diseases and disorders [2]. Several reports have proposed 14-3-3γ as a CSF biomarker for the clinical diagnosis of sporadic Creutzfeldt–Jakob disease (CJD) and several other inflammatory pathological conditions [9]. Hyperphosphorylated tau (hP-tau), a major constituent of Alzheimer’s neurofibrillary tangles, is a critical neuropathological binding target of 14-3-3ζ (zeta) [9]. 

Numerous proteins have been implicated in neurodegenerative protein aggregation; Aβ_1–42_ and hP-tau are best known as aggregate markers typical of AD, but they are also constituents of aggregates observed in other neurodegenerative diseases [10,11,12,13]. Previous research showed that tau is an interacting partner of 14-3-3Z/ζ [14]. However, the mechanisms and consequences of that interaction in AD pathology are not well-understood. In the present study, we found AD-specific interactions of 14-3-3 paralogs (G/γ and S/σ) in aggregates isolated from AD vs. control hippocampi. Proteomics of AD brain aggregates showed co-aggregation of 14-3-3 paralogs along with major seed proteins Aβ_1–42_ and tau [15]. Knockdowns of these interacting partners were evaluated for amelioration of aggregate burden and rescue of associated physiological functions. Moreover, the identified regions of proximity between 14-3-3 paralogs and their ligands can be used as therapeutic targets for disruption by protein–protein interaction inhibitors (PPIIs). We identified candidate drugs to target the interface between 14-3-3G/γ and hexokinase, its principal interacting partner, to reduce aggregation in human-cell and *C. elegans* models of neuropathic aggregation and neurodegeneration. 

## 2. Results

### 2.1. Significant Enrichment of 14-3-3 Paralogs Sigma, Gamma, and Zeta in AD 

We isolated detergent-insoluble aggregates from AD and AMC hippocampi after immunoprecipitation with antibodies to amyloid beta or tau, seed proteins implicated in AD [16]. Table 1 lists the numbers of spectral hits, showing the relative abundance of 14-3-3 paralogs in AMC and AD.

### 2.2. Molecular Dynamic Simulations and Interactome Analyses of 14-3-3 Paralogs

**Figure 1:**
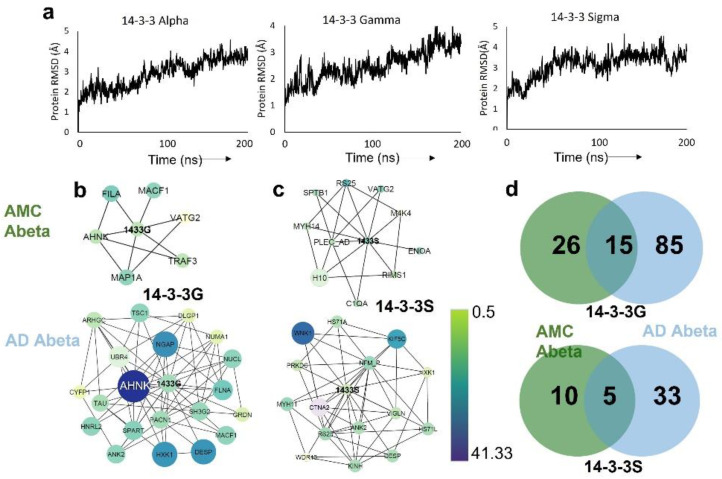
Molecular dynamic simulations and interactome analyses of 14-3-3 paralogs. (**a**) RMSD trajectories of 14-3-3 paralogs in 200-ns simulations. (**b**,**c**) Amyloid beta interactome sub-networks of 14-3-3G (**b**) and 14-3-3S (**c**) in aggregates from AMC and AD, respectively; node color indicates relative protein abundances (see key). (**d**) Venn diagram showing the 14-3-3G-interacting protein counts unique to AMC or AD, or shared in common.

Previous studies demonstrated that 14-3-3 proteins have disordered N- and C-termini as monomers but attain stable conformations when bound to a target peptide or phosphopeptide [17]. We obtained experimental structures for the conserved core regions of three of the seven mammalian 14-3-3 paralogs (γ/G/gamma, σ/S/sigma, and ζ/Z/zeta) in both the peptide-bound monomeric and dimeric states from the Protein Data Bank (PDB IDs 5N10, 4O46, and 6QHL, respectively). In order to explore the structural stability of these proteins as monomers, we conducted 200-ns atomistic molecular dynamic simulations in triplicate. Based on plots of root-mean-square deviation (RMSD) vs. time (Figure 1a), none of these 14-3-3 monomeric core regions (α/alpha, γ/gamma, and ζ/zeta) attained a stable conformation within 200 ns. 

In view of their disordered end domains, we expected the 14-3-3 proteins to bind multiple protein partners. We previously used aggregate-permeable “click” reagents to cross-link neighboring proteins within amyloid aggregates from human SY5Y-APP_Sw_ neuroblastoma cells [18] and subsequently employed the same protocol for aggregates immunopurified from AMC and AD hippocampal tissue (manuscript in preparation). In the latter study, we observed over twice as many 14-3-3 interacting partners in AD as in AMC aggregates (Figure 1b,c). Consistent with our previous work on defining aggregate interactomes [18], we found that 85–87% of AD β-amyloid proteins in close proximity to 14-3-3G or 14-3-3S were absent from AMC aggregates (Figure 1d). Several proteins interacting with 14-3-3G predominantly in AD are shown in Figure 1b,d along with their relative abundances (spectral counts) in AD vs. AMC aggregates. Our analysis of the AD interactome indicates that 14-3-3G interacts directly with tau (TAU) and other aggregate proteins, including ankyrin-3 (ANK3), plectin (PLEC), kinesin heavy chain 5C (KIF5C), and hexokinase (HXK1) (Figure 1b,c)—all of which play key roles in neurodegenerative-disease aggregation. 

### 2.3. Identification of Top 14-3-3 Interactions as Potential Targets to Disrupt Aggregation 

**Figure 2:**
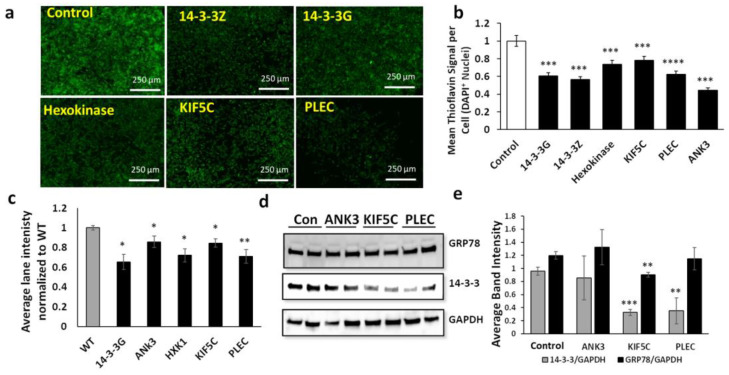
SiRNA knockdowns of influential aggregate hubs reduce aggregation in human cells. (**a**) Fluorescence images of thioflavin T-stained SH-SY5Y-APP_Sw_ cells after liposomal transfection with siRNA constructs targeting 14-3-3 paralogs or their interacting partners. (**b**) Histogram of means ± SEM for Thioflavin T staining of amyloid as in (**a**). (**c**) Intensity of SYPRO-Ruby-stained gel lanes for sarkosyl-insoluble aggregates isolated from SY5Y-APP_Sw_ cells exposed to RNAi constructs; (**d**) western blot probed with antibody to GRP78, 14-3-3, or GAPDH; gels were loaded with total protein extracted from SH-SY5Y-APP_Sw_ cells treated with RNAi constructs indicated; (**e**) mean ± SEM of band intensity for 14-3-3 or GRP78, each normalized to GAPDH. (**b**,**c**,**e**) Significance based on two-tailed *t*-tests: * *p* < 0.05; ** *p* < 0.005; *** *p* < 10^−7^; **** *p* < 10^−17^. For each assay, 100 < *N* < 150.

We assessed siRNA knockdowns targeting 14-3-3G, 14-3-3Z, and four of their most abundant interacting proteins (ankyrin, hexokinase, kinesin, and plectin) with high AD/AMC ratios and interactome influence >2, as predicted by neural-network analysis. Tau, a fifth interacting partner of 14-3-3, could not be pursued because we were unable to find an FDA-approved drug binding to the interface of these two disordered proteins. SH-SY5Y-APP_Sw_ human neuroblastoma cells were transfected with siRNA constructs specifically targeting the genes encoding each of these 14-3-3 binding partners. The impact on amyloid aggregation of each knockdown was quantified by thioflavin T staining. Total amyloid fluorescence per cell was suppressed 20–50% by these siRNA treatments (Figure 2a,b). We also quantified levels of total sarkosyl-insoluble protein recovered from these cells after acrylamide gel electrophoresis and SYPRO-Ruby staining; protein per lane was reduced 15–30% after knockdowns (Figure 2c). Total 14-3-3 protein levels in aggregates also declined after knockdowns of interacting proteins (Figure 2d) in contrast to levels of GRP78, a key mediator of the endoplasmic reticulum unfolded protein response (UPR^ER^) [19], which were not altered significantly by two of these knockdowns and only modestly (~25%) by a third (Figure 2d,e).

### 2.4. Knockdown of Key Interacting Partners of 14-3-3 Tested in Nematodes

**Figure 3:**
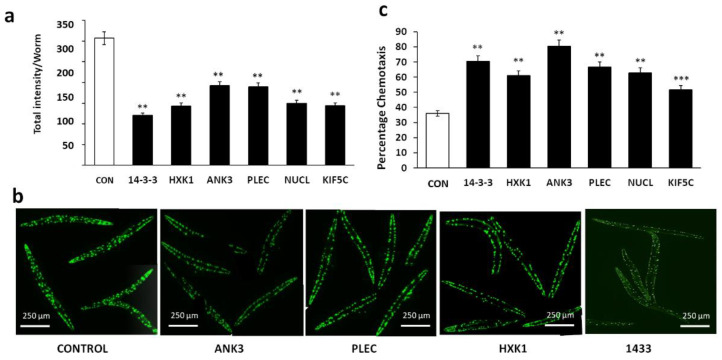
Knockdowns of influential aggregate hubs reduce aggregation in *C. elegans*. (**a**) Intensity per worm of aggregates in AM141 adults after exposure (starting in larvae) to RNAi constructs; 13 < *N* < 18. (**b**) Images of AM141 worms showing decreased size and number of aggregates after exposure to RNAi constructs. (**c**) Chemotaxis of *C. elegans* strain CL2355 adults (100 < *N* < 200) after treatment with the indicated RNAi. Significance based on two-tailed *t*-test (**a**) or Fisher exact test (**c**): ** *p* < 10^−3^; *** *p* < 10^−7^.

We next assessed the impact of RNAi-mediated knockdowns targeting the closest *C. elegans* orthologs of the above proteins in two nematode models of neurodegenerative aggregation: strain AM141, expressing a polyglutamine-array marker (Q40::YFP) in muscle cells as a model of huntingtin-like aggregation in Huntington’s disease (HD) neurons; and CL2355, which forms AD-like amyloid deposits in neurons expressing human Aβ_1–42_. In the Huntington model, total aggregate intensity per worm was reduced 40–63% by RNAi-mediated knockdown relative to empty-feeding-vector controls (Figure 3a). Worm images (Figure 3b) illustrate the decrease in both the number and fluorescence intensity (Q40::YFP content) of aggregates. We also assessed knockdowns in worms expressing neuronal β-amyloid (CL2355), which show reduced chemotaxis, a measure of chemosensory response. The decline in chemotaxis due to neuron-specific induction of a human Aβ_42_ transgene and subsequent AD-like amyloid deposition left just over a third of worms moving toward the n-butanol chemo-attractant. Knockdown of each target elicited substantial and significant rescue of chemotaxis relative to control worms, elevating their levels from 36% (FV controls) to 53–80% chemotaxis (Figure 3c). These interventions thus restored 27–70% of the deficit relative to wild-type worms (99 ± 1% chemotaxis, not shown).

### 2.5. Molecular Modeling of 14-3-3 Interactions with Key Interacting Proteins

**Figure 4:**
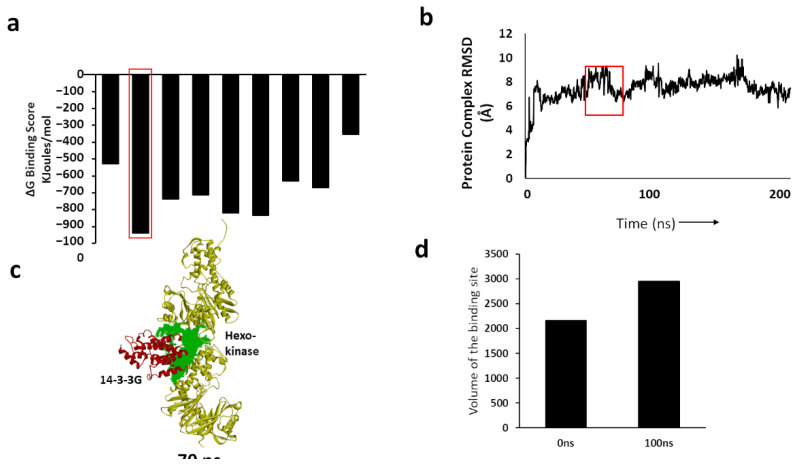
Prediction of an avid interaction between 14-3-3G and hexokinase. (**a**) Binding free energies of 14-3-3G with the principal interacting proteins. Note that hexokinase (HXK) is predicted to have the lowest free energy of binding. (**b**) RMSD plot of 14-3-3G bound to hexokinase over a 200-ns MD simulation; (**c**) 14-3-3 G::hexokinase complex showing binding pocket (green) at 70 ns. (**d**) The volume of the hexokinase binding pocket for 14-3-3G increases between 0 and 100 ns.

We performed simulations to predict structural variation that may affect aggregate adhesions of 14-3-3G/γ to either hexokinase (HXK) or kinesin heavy chains (KIF5C, KINH/KIF5B), especially the kinesin heavy chain isoform 5C, the ligand showing the top influence scores. Structures of hexokinase and kinesin were obtained from PDB (www.rcsb.org (accessed on 27 May 2021)) and I-TASSER, respectively. We performed protein–protein dockings in Hex v 8.0 (Figure 4a), which predicted far greater stability for the 14-3-3γ::hexokinase (HXK) complex (−930 kcal/mol) than for either 14-3-3γ::KIF5C (−340 kcal/mol) or 14-3-3γ::KINH (−530 kcal/mol). We simulated the 14-3-3γ::HXK complex for 200 nsec and plotted the root-mean-square deviation (RMSD) of atoms from their initial positions (Figure 4b). The RMSD time course predicted by molecular dynamic modeling suggests that the complex attains a moderately stable conformation. We selected a meta-stable “snapshot” of the complex at 70 ns and predicted its binding pocket (Figure 4c,d). 

### 2.6. In Silico Screening Identifies Drugs Predicted to Disrupt 14-3-3::Hexokinase Binding

Since the 14-3-3::hexokinase interaction is enriched in AD aggregates, we posited that disruption of their complex by a small molecule may relieve protein aggregation. Considering that both 14-3-3G and hexokinase are required for normal biological functions, targeting one or both of these proteins is likely to be toxic or to have adverse side effects. However, targeting the interface between 14-3-3 and hexokinase provides a feasible alternative that may safely oppose protein aggregation and its associated functional declines. We therefore targeted a druggable pocket created at the 14-3-3γ–hexokinase interface in the bound structure observed at 70 ns, a metastable point in the simulation when the binding pocket had begun to increase in volume (Figure 4d). To identify drugs that may disrupt the interaction between 14-3-3γ and hexokinase, we initially screened drugs from an FDA-approved library comprising >2300 compounds via in silico docking simulations. To improve screening efficiency, docking was conducted in three stages, increasing the stringency for successive screens. We began with virtual docking of the entire FDA-approved drug library, using SiBiolead to run AutoDock in high-throughput mode. The top 10% of molecules (230 drugs) were then re-docked using Glide in its standard (“high-precision”) mode. The top 10% (23 drugs) from Glide were pursued by assessing the free energy of each 14-3-3γ::HXK::drug complex in implicit solvent using the Schrödinger MM-GBSA module. The top five candidates from this third-stage analysis (Table 2) were pursued.

### 2.7. Drug Binding to Predict Disruption of the 14-3-3γ::HXK Complex 

**Figure 5:**
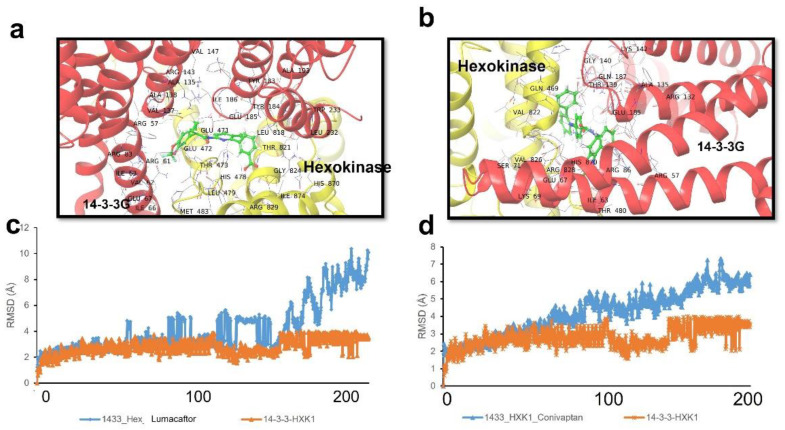
Protein aggregation or its sequelae are reduced by drugs that target the 14-3-3G::hexokinase interface. (**a**,**b**) Predicted binding sites of repurposed-drug candidates lumacaftor (**a**) and conivaptan (**b**), at the interface between hexokinase and 14-3-3G. (**c**,**d**) RMSD plots of the 14-3-3G:: hexokinase complex, as predicted in the absence (orange) or presence (blue) of lumacaftor (**c**) or conivaptan (**d**), simulated over 200-ns timespans.

The above FDA-approved drugs were docked to the 14-3-3::HXK binding pocket in atomistic molecular dynamic simulations. The predicted binding sites and poses of the top-ranked two drugs (lumacaftor and conivaptan) are shown in Figure 5a,b. The ability of these drugs to disrupt the interaction between 14-3-3γ and human hexokinase (HXK) was predicted using molecular-dynamic simulations. Figure 5c,d depicts the RMSD during a 200-nsec simulation for the 14-3-3G::HXK complex, with or without binding of lumacaftor or conivaptan at the protein–protein interface. The RMSD plots illustrate progressive expansion of the 14-3-3G::HXK interaction complex in the presence of either drug relative to the 14-3-3::HXK complex alone. These predictions support pursuit of the drugs conivaptan and lumacaftor as candidates to disrupt the 14-3-3::HXK interaction and thereby relieve protein aggregation.

### 2.8. Top-Ranked Drugs Rescue C. elegans and Human-Cell Aggregation Models

**Figure 6:**
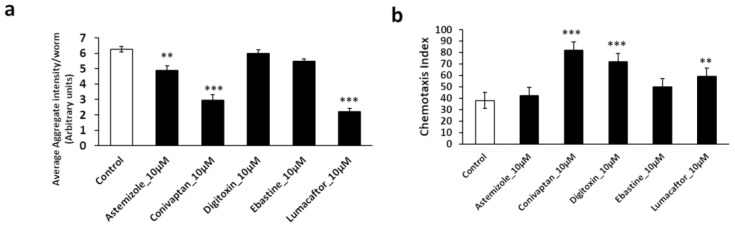
Drugs targeting the hexokinase::14-3-3 interface reduce aggregation in *C. elegans* models of human neuropathology. (**a**) Calculated average aggregate counts per worm in *C. elegans* strain AM141 (*q40::yfp*), a model of HD in which muscle expression of polyglutamine (Q40) fused in-frame to the gene encoding yellow fluorescent protein (YFP) leads to age-dependent accrual of yellow fluorescent aggregates. YFP-positive aggregates were counted in adult worms on day 5 post-hatching; significances were determined by two-tailed *t*-tests with 13 < *N* < 18. (**b**) Chemotaxis index of *C. elegans* strain CL2355, in which uninduced pan-neuronal expression of human Aβ_1–42_ causes age-dependent loss of chemotaxis [15,16], here assessed on day 5 post-hatching. Significances were determined by Fisher exact tests (100 < *N* < 200): ** *p* < 0.005; *** *p* < 0.0005.

All five top-ranked drugs (Table 2) were tested for rescue of protein aggregation in *C. elegans* strain AM141 (a model of polyglutamine aggregation in Huntington’s disease). The total intensity of aggregates was assessed after exposure of nematodes to each drug at two concentrations. The top two drugs (conivaptan and lumacaftor, each at 10 μM) reduced protein aggregation by 60–70% (Figure 6a). A third drug, asfemilzole at 10 μM, reduced aggregation ~25%. We also tested these drugs in a *C. elegans* model of AD-like amyloidosis expressing neuronal Aβ_1–42_ and consequently suffering impaired chemotaxis. Chemoattraction to n-butanol was 38.5% for untreated worms (vs. 99 ± 1% for worms not expressing Aβ_1–42_), but restored to 82% by conivaptan, 72% by digitoxin, and 60% by lumacaftor (each at 10 μM; Figure 6b). 

**Figure 7:**
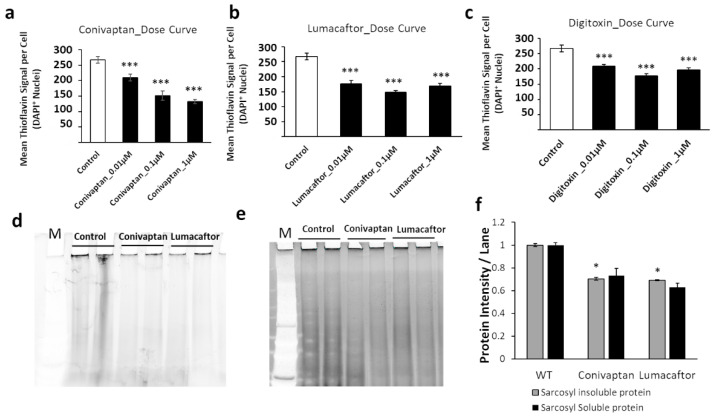
Drug effects in human cells. (**a**–**c**) Dose–response curves for SY5Y-APP_Sw_ cells treated with three top-ranked drugs at 0.01, 0.1, or 1 µM. Cells were stained with thioflavin T and DAPI to quantify amyloid per cell. Significances according to two-tailed *t*-tests (100 < *N* <150): *** *p* < 10^−5^. Sarkosyl-insoluble (**d**) and sarkosyl-soluble aggregates (**e**) from SY5Y-APP_Sw_ cells were resuspended in Laemmli buffer at 95 °C and electrophoresed on acrylamide gels. Gels were stained with SYPRO-Ruby and protein quantified as fluorescence per lane. (**f**) Histogram shows sarkosyl-insoluble and sarkosyl-soluble aggregate proteins from gels such as those illustrated in panels (**d**,**e**). Significances based on one-tailed heteroscedastic *t*-tests (*N* = 4): * *p* ≈ 0.01.

These drugs were next tested on human neuroblastoma (SH-SY5Y-APP_Sw_) cells exposed to each drug for 48 h at three concentrations (0.01, 0.1, and 1 μM). Conivaptan at 1 μM reduced amyloid fluorescence per cell by 52% as quantified by thioflavin staining, while lumacaftor achieved a 48% reduction, and digitoxin a 35% reduction, each at 0.1 μM (Figure 7a–c). We pursued these results by isolating aggregates from SY5Y-APP_Sw_ cells treated with 0.1 μM conivaptan or lumacaftor, which reduced sarkosyl-insoluble and -soluble aggregates 30–40% and 35–45%, respectively (Figure 7d–f). 

### 2.9. Hexokinase Is Recovered from Neuroblastoma Cells by 14-3-3 Immuno-Pulldown 

**Figure 8:**
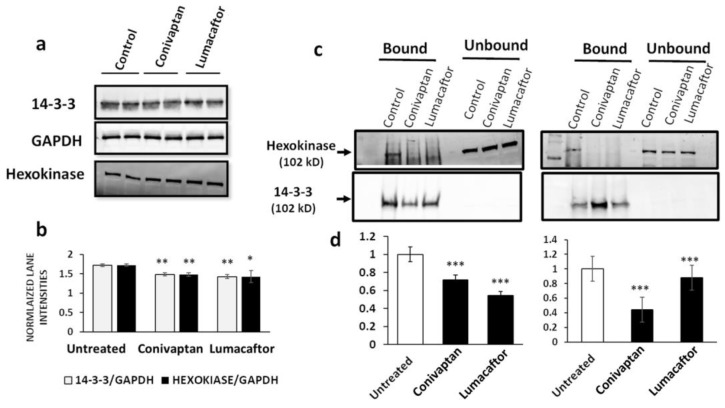
Hexokinase interacts with one or more 14-3-3 proteins in human neuroblastoma cells. (**a**) Acrylamide gels were loaded with 50 μg total protein per lane extracted from SH-SY5Y-APP_Sw_ cells after a 48 h exposure to FDA-approved drugs lumacaftor or conivaptan. Western blots were probed with antibodies to the 14-3-3 conserved core, hexokinase, or GAPDH. (**b**) Mean ± SD band intensities of hexokinase lanes (*N* = 3) normalized to the corresponding 14-3-3 band. (**c**) Antibody to the 14-3-3 conserved core was used for immunopulldown of 14-3-3 complexes from neuroblastoma cells either treated for 48 hr with drugs or untreated. Gel lanes were loaded with protein recovered from coated magnetic beads and electrophoresed, and western blots were probed to detect hexokinase or 14-3-3 proteins. (**d**) Histogram of hexokinase recovery (signals on western blots as in (**c**)), normalized to 14-3-3; the band signal is plotted for mean ± SD band intensity ratios of replicates. Significances according to two-tailed heteroscedastic *t*-tests (**b**,**d**), each *N* = 4: * *p* = 0.05; ** *p* < 10^−2^; *** *p* < 10^−3^.

We then asked whether human neuroblastoma (SH-SY5Y-APP_Sw_) cells expressing the APP_Sw_ double mutant observed in familial AD [20] contain aggregates in which any 14-3-3 protein interacts with hexokinase. We first quantified 14-3-3 paralogs and hexokinase in cell lysates to ensure that their levels were not severely depleted by the drug. This appeared to be the case (Figure 8a,b); although several treatment groups differed significantly from controls, the difference was <20%. We used a co-immunopulldown (co-IP) strategy to recover and quantify binding of 14-3-3 to hexokinase. Complexes or aggregates isolated by IP using magnetic beads coated with antibody to the conserved 14-3-3 core were recovered, resuspended in hot loading buffer, and electrophoresed on acrylamide gels, and their western blots were probed with antibody to hexokinase or the 14-3-3 conserved core. Co-IP of hexokinase (normalized to 14-3-3 recovery) was reduced >45% by treatment with drugs specific to the binding of the interface between 14-3-3 and hexokinase (Figure 8c,d). 

## 3. Discussion

Previous studies have documented the critical roles played by 14-3-3 proteins in diverse neurological and other age-associated disorders [1,2,5]. In eukaryotes, 14-3-3 paralogs are conserved adapter proteins involved in multiple physiological processes, such as signal transduction, translation, protein trafficking, and apoptosis [21]. Deficiencies in specific 14-3-3 paralogs in knockout mice result in neurotransmitter imbalance and altered behavior that has been likened to schizophrenia [22]. 

In the current study, we used computational methods to predict disordered regions of 14-3-3 paralogs that fail to attain stable conformations on their own, resulting in indeterminate (or partner-determined) structures. These paralogs are rich in basic amino acid residues, aromatic amino acids, and amphipathic amino acids relative to acidic amino acids. We observed an increased sequestration of 14-3-3 proteins into sarkosyl-insoluble aggregates during aging and also in disease states both in heart and brain [15,23,24]. In order to target protein–protein interaction interfaces for the treatment of neurodegenerative diseases, including Alzheimer’s disease, we proposed to use small molecules as protein–protein interaction inhibitors to counteract aggregate progression by breaking critical interactions needed for aggregate growth. 

We first identified 14-3-3 interacting partners by performing crosslinking analysis of hippocampal aggregate proteins isolated from Alzheimer’s disease vs. age-matched controls. A total of 85 interacting proteins were associated with 14-3-3 only in tissue from AD, whereas only 26 protein partners were unique to controls. Previous studies showed that 14-3-3θ (theta) acts as a chaperone to assist in refolding of disordered proteins, such as α-synuclein, thus reducing its toxicity; 14-3-3θ-overexpressing mice are protected from toxic effects of α-synuclein fibrils [25]. 

AD is characterized by tau hyperphosphorylation, contributing to elevated tau in paired helical filaments (PHF) and tangles in neurons [26]. Prior studies showed 14-3-3 interaction with hyperphosphorylated tau (hP-tau), preventing access to phosphatases and, thus, indirectly promoting its aggregation and PHF formation [14]. Several 14-3-3 paralogs were also shown to interact with polyglutamate (polyQ), and 14-3-3ζ (zeta) plays an essential pro-aggregation role in a cell culture model of Huntington’s disease [27]. 

Since disruption of 14-3-3 protein results in the development of diabetic cardiomyopathy [28] and neurodegeneration [22,25,29,30,31], we used an approach that targeted protein–protein interactions unique to the disease state. Pharmacological disruption of protein–protein interactions should not affect the normal biological functions mediated by either of the interacting proteins, thus holding the promise of very limited side effects. The validity of our approach was supported by the screening of an FDA-approved drug library, leading to the discovery of existing drugs that could be repurposed to prevent specific pro-aggregative interactions of 14-3-3 paralogs (See Supplementary Figure S1). This provides an express route to novel therapeutics available immediately but, at the same time, offers the promise that screening of large structural libraries of small molecules is likely to lead to even better drug candidates with highly specific (and non-essential) targets. Such screens will be the subject of a forthcoming paper. 

In conclusion, we demonstrated that drugs targeting the interfaces of 14-3-3 paralogs with their interacting partners show promise for the reduction of aggregation and the improvement of associated physiological functions. Such disease-specific protein–protein interaction inhibitors have the potential to prevent, slow, or reverse aggregation associated with neurodegenerative diseases and other age-progressive disorders. 

## 4. Materials and Methods

### 4.1. C. elegans Strains 

Transgenic *C. elegans* strains used in this study were obtained from the Caenorhabditis Genetics Center (CGC; Minneapolis, MN, USA), and maintained at 20 °C on 2% (*w*/*v*) agar plates containing 1× nematode growth medium and overlaid with a central lawn of *E. coli* strain OP50 (unless otherwise noted). The nematode models of neuropathic aggregation used in this study were: (*i*) strain CL2355 (dvIs50 [pCL45 (snb-1::Aβ1-42::3′ UTR(long) + mtl-2::GFP]), which drives pan-neuronal expression of human Aβ_1–42_ peptide; and (*ii*) AM141 (rmIs133 [unc-54p::Q40::YFP]), in which Q40::YFP synthesis in body wall muscle cells serves as a model of protein aggregation dependent on expansion of polyglutamine (polyQ) arrays, as occurs in huntingtin protein resulting in amyloid-aggregate deposition. 

### 4.2. Chemotaxis Assays in an Aβ-Transgenic C. elegans Strain

Transgenic *C. elegans* expressing Aβ_1–42_ in neurons (strain CL2355) were grown at 20 °C with ample *E. coli* (OP50) bacteria, and adult worms were lysed to release unlaid eggs and, thus, to initiate a synchronized-aging cohort. Eggs were then placed on 100 mm NGM-agar Petri dishes seeded with RNAi-expressing bacteria (strain HT115) targeting orthologs of some of the interacting partners of 14-3-3 specific to AD or empty-vector control bacteria. Worms at the L3–L4 transition were upshifted to 25.5 °C to induce human Aβ_1–42_ transgene expression and assayed after 48 h. Alternatively (Figure 6), worms were aged to adult day 5 without upshift. Chemotaxis-to-butanol and paralysis assays were performed as described previously [15,16,24,32,33]. 

### 4.3. RNAi in C. elegans 

Gene knockdowns were achieved through RNA interference (RNAi) by feeding dsRNA-expressing bacteria as described [24,32,34]. Briefly, synchronized worms were placed, either immediately after hatching or at the late-L4 larval stage, onto IPTG-containing NGM plates seeded with bacteria (*E. coli* HT115[DE3]) carrying the empty L4440 vector (pPD129.36) or with similar bacterial clones expressing double-strand RNA corresponding to exonic regions of *unc-44* (encoding an ortholog of human ANK3), *vab-10* (PLEC), *exc-7* (NUCL), or *unc-116* (KIF5C). Day-3 adult worms were imaged for aggregate count, assessed for paralysis, or evaluated for chemotaxis toward n-butanol.

### 4.4. siRNA Knockdowns of Human Cells and Thioflavin T Staining to Quantify Amyloids

Exponentially growing cultures of SY5Y-APP_Sw_ (human neuroblastoma) cells were trypsinized and replated at 8000–10,000 cells/well in 96-well plates and grown for 16 h at 37 °C in DMEM + F12 (Life Technologies; Carlsbad, CA, USA) medium supplemented with 10% (*v*/*v*) fetal bovine serum. When cells reached ~40% confluence, siRNAs were transfected by lipofection (RNAiMax, Life Technologies; Carlsbad, CA, USA) to target genes encoding ANK3 (SAS1_Hs_00065571), YWHAZ (SASI_Hs01_00210839), YWHAG (SASI_Hs01_00201711), KIF5C (SAS1_Hs_ 00065571), PLEC (SAS1_Hs_00039321), or NUCL (SAS1_Hs_00217223), all obtained from Millipore-Sigma (St. Louis, MO, USA) and used as directed by the manufacturer. Transfected cells were assayed for protein aggregation 48 h later by fixation in 4% formaldehyde and staining with 0.1% *w*/*v* Thioflavin T. After four washes in PBS, cells were covered with Antifade + DAPI (Life Technologies) and fluorescence was captured in both blue and green channels (Keyence fluorescence microscope with motorized stage) for automated well-by-well imaging, with nine fields per well. Thioflavin T fluorescence intensity was divided by the number of DAPI^+^ nuclei to obtain normalized values (amyloid fluorescence per cell), summarized as means ± SD. 

### 4.5. Western-Blot Analysis of SH-SY5Y-APP_Sw_ Aggregate Proteins for 14-3-3, GRP78, GAPDH, and Hexokinase

Human SH-SY5Y-APP_Sw_ neuroblastoma cells were maintained in cell culture medium (DMEM; Invitrogen/Life Technologies, Grand Island, NY, USA) supplemented with 10% *v*/*v* fetal bovine serum (FBS). After treatment with either shRNA or a drug candidate, cells were harvested and their proteins extracted in lysis buffer containing 50-mM Tris-HCl (pH 7.5), 150-mM NaCl, 1% *w*/*v* Nonidet P40, 0.1% SDS, and 0.5% sodium deoxycholate. Protein was quantified with Bradford reagent (Bio-Rad; Hercules, CA, USA), and 50 μg protein aliquots were electrophoresed for 2 h at 100 V on 4–20% gradient bis-tris acrylamide gels (BioRad Life Science, Hercules, CA, USA) and transferred to nitrocellulose membranes. Blots were blocked with BSA blocker (Pierce) and incubated overnight at 4 °C with primary antibodies to 14-3-3 (ThermoFisher, Waltham, MA, USA, 14-3-3 Pan Antibody, 1:5000 dilution), GRP78 (ThermoFisher, diluted to 2 μg/mL; Waltham, MA, USA), GAPDH (ThermoFisher, 1:2000 dilution; Waltham, MA, USA), or hexokinase (ThermoFisher, 1:1000 dilution; Waltham, MA, USA). After four washes of 6 min each, membranes were incubated for 1 h at ~20 °C with HRP-conjugated secondary antibody—goat anti-rabbit IgG (AbCam, 1:10,000 dilution; Waltham, MA, USA), rabbit anti-goat IgG (Rockland Immunochemicals, Gilbertsville, PA, USA; 1:10,000 dilution), or goat anti-rabbit IgG (AbCam, 1:3000 dilution; Waltham, MA, USA)—and developed using an ECL chemiluminescence detection kit (Pierce). Data were digitized and analyzed using ImageJ software (NIH). 

### 4.6. Isolation of Protein Aggregates 

Human cells treated with siRNA or drugs were collected, flash frozen in liquid nitrogen, and homogenized on ice in buffer containing nonionic detergent (1% *v*/*v* NP40, 20 mM HEPES (pH 7.4), 300 mM NaCl, 2 mM MgCl_2_, and protease/phosphatase inhibitors (CalBiochem; San Diego, CA, USA). Lysates were centrifuged at 3000 rpm for 5 min at 4 °C to remove debris. Protein was sonicated to disrupt cell membranes and membrane-bound organelles. Following removal of cytosolic proteins (soluble in 1% NP40 nonionic detergent) by centrifugation (18 min, 13,000× *g* at 4 °C), protein pellets were brought to pH 7.4 with 0.1 M HEPES buffer containing 1% *v*/*v* sarkosyl (sodium lauryl sarcosinate) and 5 mM EDTA, heated to 95 °C for 10 min, and centrifuged for 30 min at 100,000× *g*. Sarkosyl-insoluble proteins (pellet fraction) were resuspended in Laemmli loading buffer (containing 50 mM dithiothreitol and 2% *v*/*v* sodium dodecyl sulfate (SDS)), heated for 5 min at >95 °C, and resolved by electrophoresis on 4–20% polyacrylamide gradient gels with 1% SDS. Gels were stained with SYPRO-Ruby (ThermoFisher; Waltham, MA, USA) or Coomassie Blue (ThermoFisher; Waltham, MA, USA) to visualize protein. 

### 4.7. Molecular Dynamic (MD) Simulations of 14-3-3 Paralogs 

The X-ray crystallographic structures of 14-3-3 paralogs (14-3-3S/σ, 14-3-3G/γ, 14-3-3Z/ζ) were obtained from the PDB database (www.rcsb.org; accessed on 25 May 2021) or modeled using I-TASSER (https://zhanggroup.org/I-TASSER/ accessed on 2 June 2021), an online server that uses fold-recognition and ab initio modeling [35,36]. To understand the dynamic behavior of protein structure, target proteins interacting with 14-3-3 paralogs (e.g., hexokinase for 14-3-3G) were simulated using Schrödinger Maestro (version 11.9.011). Each protein went through a preparation stage of preprocessing with default parameters, including pH, using Maestro protein preparation wizard, after which an orthorhombic box was created around the protein with a minimized volume that varied with the protein. The system was first neutralized using the appropriate salt concentration (Na^+^, Cl^−^), and a further 0.15M NaCl was added to the system to mimic the physiological conditions. The temperature and pressure were held constant (300 °K; 1.1023 bar). A random seed number was entered prior to starting each simulation, which was maintained for 200 ns (or as indicated) and replicated ≥3 times with new seeds.

### 4.8. MD Simulation Identifies a Druggable Target for 14-3-3G Interaction with Hexokinase 

Docking studies were conducted using Hex, an interactive protein-docking and molecular-superposition program. Hex identifies the most stable interaction of two proteins (i.e., with the most negative ΔG). The complex of 14-3-3G with hexokinase was exported and simulated using Schrödinger Maestro. After MD simulation, the complex was captured, usually at an RMSD “plateau”. The druggable pocket or interface was determined using the Discovery Studio Receptor Cavities plug-in to predict likely druggable sites. 

### 4.9. High-Throughput Computational Screen to Identify Novel PPII Molecules to Disrupt the 14-3-3G–Hexokinase Interaction 

The interface at which 14-3-3G and hexokinase interact was screened against two structural drug libraries, each at three successive stages of increasing stringency. The FDA-approved drug library (https://www.fda.gov/drugs accessed on 8 June 2021) was prepared using Biovia Discovery Studio and was virtually screened as follows. The first screen was conducted with SiBiolead (www.sibiolead.com accessed on 12 June 2021) running high-throughput screening with AutoDock. This program accepts proteins in PDB format as inputs, along with amino acid numbers that define a grid box around the targeted binding region. The user specifies the library to screen and initiates the search. The time to completion depends on the sizes of the library and the protein. 

We retrieved the top 10% of drugs from the first-stage high-throughput docking screen as inputs for second-stage docking at higher stringency. This stage used high-precision Glide docking implemented within Schrödinger Maestro. Inputs for Glide docking included a protein specified in PDB format and ligands in *.mae format. To convert ligands in the library from *.mol2 to *.mae format, we used the Ligprep plug-in under Maestro, which works in “batch” mode, returning ligands as a single file in *.mae format. Glide docking predicts binding poses for a drug with high precision. Grid boxes were formed around the receptor region by specifying amino acid residue numbers; default values were used for all other parameters. 

The top 10% of drugs from the second-stage screen were advanced to stage three, in which we used the MM-GBSA plug-in (molecular mechanics with generalized Born surface area) from Schrödinger Maestro to predict binding free energies for all ligands based on inputs from Glide docking, employing core and ligand settings. The output lists top candidates for specific binding to a target, which can be either a protein or a protein–protein interface (Supplementary Figure S1). 

### 4.10. Statistical Analyses

For assays in which *N* was <10, differences between control and experimental groups were assessed for significance by the Fisher-Behrens heteroscedastic *t*-test (appropriate to samples of unequal or unknown variance) [37]. In some cases, the significance of experimental reproducibility was also evaluated in this way, treating each experiment as a single point. For *N* > 10, two-tailed *t*-tests were utilized unless 1-tailed tests were justified by previous results. Within experiments, differences in proportions (fractional paralysis or chemotaxis) were assessed by chi-squared or Fisher exact tests, as appropriate based on sample counts. For assays with multiple end-points, the threshold for significance was adjusted to *p* < 0.01 to reduce the frequency of type I errors.

## 5. Patents

A patent application is in preparation for the drugs described herein.

## Figures and Tables

**Table 1:** Spectral hits for three paralogs of 14-3-3 in AD and AMC aggregates, isolated by immunoprecipitation (IP) with antibodies to seed proteins Aβ_1–42_ or tau, or “total” aggregates without IP.

Protein	AD Aβ_1–42_ Aggregates	AD Tau Aggregates	Total AD	AMC Aβ_1–42_ Aggregates	AMC Tau Aggregates	Total AMC
14-3-3S	57	50	242	4	6	18
14-3-3G	58	60	234	9	2	27
14-3-3Z	71	67	302	3	0	18

**Table 2:** Top drug candidates are shown, along with their binding scores and the conditions they were FDA-approved to treat.

Drug Name	Binding Score	Drug Type/Conditions Treated
Conivaptan	−11.4	Low blood sodium
Lumacaftor	−10.6	Protein folding chaperone; increases CFTR proteins
Ebastine	−10.2	H_1_ antihistamine with low narcolepsy potential
Digitoxin	−10.1	Low-output congestive heart failure
Astemizole	−10	Antihistamine used to treat allergies

## Data Availability

Full data will be made available to the research community upon request.

## Supplementary Materials

The following supporting information can be downloaded at: https://www.mdpi.com/article/10.3390/ijms232415457/s1, Figure S1: FDA-approved drug screening.
