# Supplementary figures and images for: Physiological Consequences of Targeting 14-3-3 and Its Interacting Partners in Neurodegenerative Diseases
Source: Int J Mol Sci. 2022 Dec 7;23(24):15457. doi: 10.3390/ijms232415457 (PMC9779020; doi:10.3390/ijms232415457)

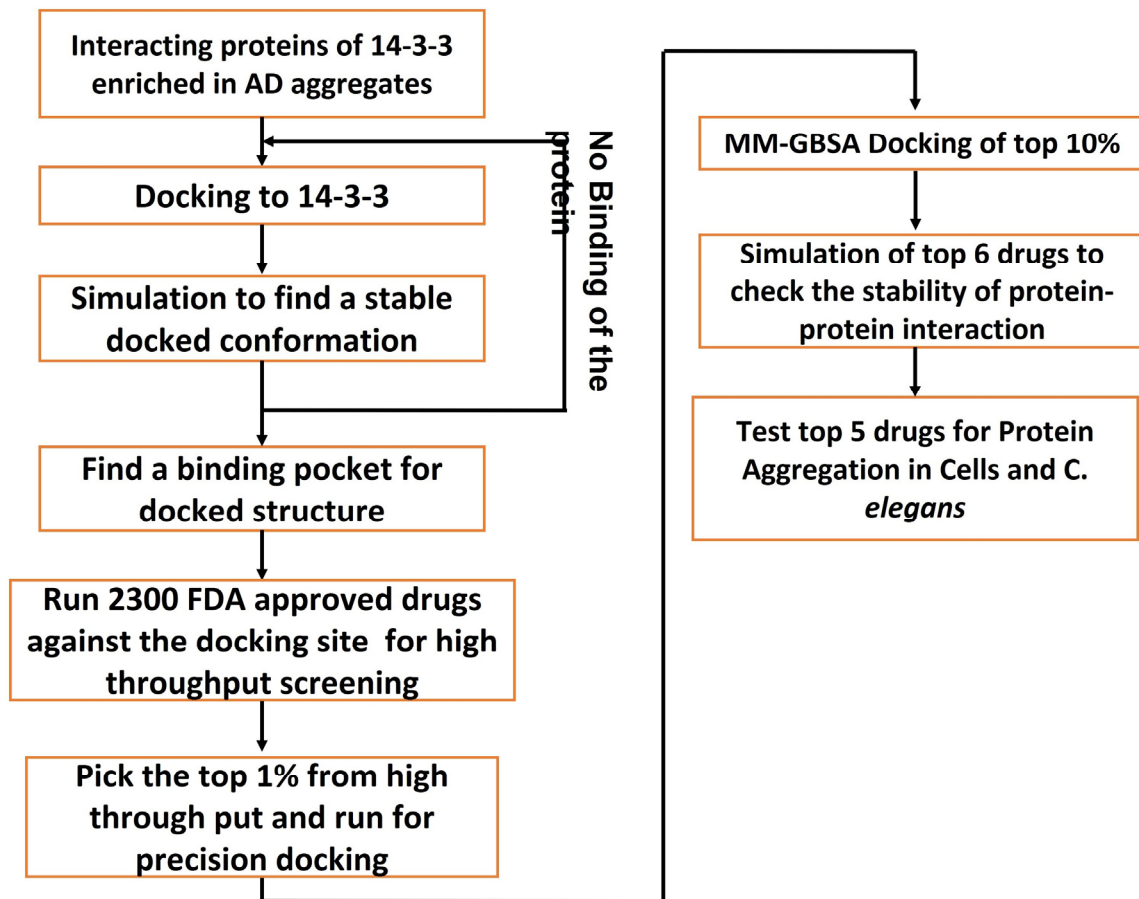

Figure S1. FDA-approved drug screening.

Supplement: Supplementary file 1 [file ijms-23-15457-s001.zip › ijms-2029945-supplementary.pdf]
